# Balancing Public & Economic Health in Japan during the COVID-19 Pandemic: A Descriptive Analysis

**DOI:** 10.3390/epidemiologia3020016

**Published:** 2022-04-08

**Authors:** Gainha Kim, Justine M. Natuplag, Sui Jin Lin, Jinyi Feng, Nicolas Ray

**Affiliations:** 1 Graduate Institute of International and Development Studies (IHEID), Chemin Eugène-Rigot 2, CH-1202 Geneva, Switzerland; 2 Institute of Global Health, University of Geneva, 9 Chemin des Mines, CH-1202 Geneva, Switzerland; justine.natuplag@etu.unige.ch (J.M.N.); nicolas.ray@unige.ch (N.R.); 3 Geneva School of Social Sciences, University of Geneva, Uni Mail, Boulevard du Pont-d’Arve 40, CH-1205 Geneva, Switzerland; sui.jin-lin@etu.unige.ch (S.J.L.); jinyi.feng@etu.unige.ch (J.F.); 4 Institute for Environmental Sciences, University of Geneva, Boulevard Carl-Vogt 66, CH-1205 Geneva, Switzerland

**Keywords:** 2020 Tokyo Olympics, Go to Travel Campaign, Japan COVID-19 policies, Shinzo Abe, Diamond Princess Cruise, Japanese politics

## Abstract

Despite loose restrictions and a low mortality rate due to COVID-19, Japan faced the challenge of stabilizing its economy during the pandemic. Here, we analyzed how the Japanese government attempted to maintain a balance between the health of the population and the health of the economy. We used a mix of quantitative data, information from policy documents, and news agency publications. Features of the Japanese government’s handling of the pandemic include the lack of constitutional authority to enforce a lockdown, the laxer restrictions compared with other countries in which citizens were advised only to exercise self-restraint and avoid close social contact, and the existence of expert panels that had only an advisory role. Our findings address the slow initial response of the government, which feared that the 2020 Tokyo Olympics would be canceled, and the increased testing when the Olympics were postponed, as well as the expansion of vaccination efforts after the Olympics. In addition, there was a targeted campaign to promote national travel to increase economic revenue in the tourism sector, but this led to an increase in COVID-19 cases.

## 1. Introduction

On 30 January 2020, the World Health Organization (WHO) officially announced the formal assertion of the COVID-19 outbreak, the disease caused by the SARS-CoV-2 virus, as a Public Health Emergency of International Concern (PHEIC) [1]. WHO’s announcement was a month after the Chinese government reported the COVID-19 outbreak in Wuhan. In the presence of asymptomatic infections, the Chinese government imposed non-pharmaceutical measures, including quarantine, isolation, physical distancing, and restrictions of movement [2]. The measures reflected the maximum 14-day incubation period of the virus, which assisted in preventing further infection by those who were asymptomatic [3].

Despite these measures, the effects of globalization fueled the rapid circulation of the virus. It soon transmitted to other countries in Asia and subsequently to every other continent [4]. The COVID-19 pandemic has presented political, economic, and public health challenges to governments across the globe. Many countries have enforced restrictive policy measures to suppress the transmission of the virus. Countries such as the United Kingdom, Italy, and France imposed a lockdown that lasted for months. A lockdown is defined here as suspending all non-essential activities [5], which reduces people’s mobility to services such as schools and workplaces. With a lockdown, a mandate to stay at home is usually enforced, except in certain pre-defined circumstances [6]. The underlying goals of COVID-19 related policies were to protect the health of the population while suppressing further infections and avoiding an overload of health care systems [7]. Not only does a lockdown delay risk of transmission by reducing contact, but it also acts as an indicator for the population to swiftly change their behavior.

Imposing a lockdown has proved to be an effective policy measure to prevent the emergence of further infections and to contain the virus within an area. For example, Northern Italy introduced a swift lockdown measure on 8 March 2020, which contributed to the spread of the virus to the southern part of the country. A lockdown has also proven to be effective to decrease the R value, and research results show that thousands of lives could have been saved by imposing an earlier lockdown in the US [5,8]. Enforcing a lockdown also allows the governments to save more time to identify the core regions and prepare systematic testing [9].

However, implementing a lockdown comes with unavoidable costs to a country’s economy that may have a negative impact in the future [10]. For example, Aum et al. (2020) estimated that half of the job losses could be attributed to lockdown in the US and the UK [11]. Miles et al. [6] concluded that the cost of three months of lockdown in the UK was higher than its benefit.

During the COVID-19 pandemic, many governments had to make a delicate decision between the good of public health and economic impact [4]. The response to the COVID-19 pandemic worked as a governance assessment tool for each country. Governments were mandated to curb the infection in a timely and efficient manner by providing the appropriate resources to their health care systems and imposing restrictions whilst considering the economic impact at the same time [4]. Moreover, the magnitude of the impact of COVID-19 on countries is heavily intertwined with political decision-making [12]. To minimize the socio-economic disruption, governments introduced COVID-19 specific economic stimulus policies, such as temporary exemption from value-added taxation [4].

Thus far, without a lockdown, Japan has mitigated the number of COVID-19 cases and mortality rate efficiently despite its large population and its proximity to China [13]. Japan’s elderly population made it difficult to keep mortality rates low, with 29% of the population aged 65 and older. Moreover, Japan’s life expectancy age is approximately 85 years, the second-highest in the world [14]. This contradicts earlier studies showing that there is usually a tendency of having a stricter lockdown in countries where the aging population is numerous [7]. One peculiarity of Japan in terms of pandemic responsiveness is that the national government does not have the constitutional power to enforce a lockdown [15]. Therefore, imposing a lockdown has not been possible in Japan. Instead, the government requested citizens to practice self-restraint and recommended avoiding the “3Cs”—Closed place, Crowded space, Close contact setting. Moreover, social distancing and wearing a face mask were social norms in Japan even before the COVID-19 pandemic.

There are several studies related to Japan in response to COVID-19, but these studies lack in-depth exploration of what factors influenced the Japanese government to make pandemic-related decisions. The study of these factors is particularly interesting given the political turmoil surrounding the resignation of prime ministers and the postponement of the Tokyo Olympics. Moreover, the studies emphasized the difference between Japanese culture from that of the West as the main analysis [10,13,15]. Tashiro et al. [13] attribute the “Japanese paradox” of loose restrictions and a low mortality rate to the cultural habits of the population, such as greeting without physical contact and the everyday wearing of masks. In addition, handwashing with soap and water is essential in early-age Japanese education, with special emphasis on the practice before meals [13]. Articles and media mainly focused on the fact that Japan’s mild lockdown has worked, and not on the process behind the government’s decision to impose a milder restriction in comparison to other countries [15]. As Inoue et al. suggest, Japan has multiple distinctive factors that contributed to its low mortality rate and controlled outbreak, including its national politics [10].

Despite the success of the “Japanese paradox”, Japan was unable to avoid political turmoil [10]. Prior to the COVID-19 pandemic, Japan enjoyed decade-long political stability. At the beginning of the pandemic and still at the time of writing, the Liberal-Democratic Party (LDP) held the majority power in Japan. The LDP is Japan’s largest political party and has held majority power in the country since the party’s establishment in 1955. The party is characterized as conservative and pro-business [16]. The last three prime ministers of Japan—Shinzo Abe (2012–2020), Yoshihide Suga (2020–2021), and Fumio Kishida (2021–present)—are part of the LDP. The onset of the COVID-19 pandemic impacted the Abe administration negatively. The administration faced accusations of bribery and illicit use of political funds for cherry blossom-viewing parties at a time when the government was recommending social distancing (Sugiyama, 2020) [17]. For the Japanese economy, the pandemic posed several challenges to the Abe administration’s plans, especially regarding boosting inbound tourism [18].

As a result of the COVID-19 pandemic, the country experienced economic and political hardship. A key example was the decision to proceed with the 2020 Tokyo Olympics. The cancelation of the 2020 Tokyo Olympics would have meant an estimated loss of 4.5 trillion Japanese yen for the Japanese government and its partners [19]. The Olympic games were postponed until July 2021, and more than 80% of the Japanese people did not favor hosting the event [20]. To limit COVID-19 infections, the Japanese government aimed to vaccinate as many citizens as possible ahead of the 2020 Olympics with the goal of vaccinating all 36 million older adults by the end of July [21]. The government established other guidelines to limit infections during this period, such as asking bars and restaurants to close entirely or by 8 pm to curb close contact socialization [22].

Approximately 10% of the global Gross Domestic Product (GDP) of Japan is derived from the tourism and hospitality industry [23]. Similar to other countries, Japan also experienced economic hardship as a result of the decrease in tourism revenue. The Japanese government intermittently halted issuing new visas and closed its borders to foreign tourists. Tourism plays a significant role in the Japanese economy. In 2019, there were 31.88 million foreign visitors. This represented 4.81 trillion yen in tourism expenditures. Spending by domestic travelers also increased from 21.1 trillion yen in 2017 to 21.9 trillion yen in 2019. In 2020, 95% of Japan’s tourism GDP came from domestic visitor expenditure [24]. The Japan Tourism Agency reported that the number of overnight hotel stays significantly decreased when compared to the same period in the previous year, from March to June 2020 [25]. To compensate for this economic loss, the Abe administration launched the “Go to Travel” national travel campaign [18]. The program was characterized by subsidies covering 50% of travel expenses, up to 20,000 yen per person or 10,000 yen per day trip [26].

Although COVID-19 had a notable impact on Japanese politics and its national events, there have been few studies that connect the political situation and public health policy decision-making. The purpose of this study is to explore the political dynamics of Japan and describe how the Japanese government has thus far attempted to maintain a balance between the health of the population and the health of the economy amidst the political turmoil during the COVID-19 pandemic.

## 2. Methodology

To examine how the Japanese government balanced public and economic health, we examined national-level policies and guidelines and their respective impacts on the country’s COVID-19 cases, looking in particular at the Japanese government’s initial response to the pandemic, the Go to Travel campaign, and the 2020 Tokyo Olympics. We aimed to provide insight into the decisions of the Japanese government by analyzing the dynamics behind political milestones during the COVID-19 pandemic. Most publications on Japanese politics are written in Japanese. Therefore, we also seek to translate key pieces of information from these publications into English to help a wider audience understand the mechanisms and decision making in Japan during the pandemic. Our research was conducted using quantitative COVID-19 epidemiological data, policy data from government agencies, and data from three impactful events in Japan during the pandemic.

Quantitative COVID-19 epidemiological data were used to understand the general situation of the country. This data adds value to this work because the government agency that tracks and reports on COVID -19, the Japanese Ministry of Health, Labour, and Welfare, does not provide digital daily chronological data on COVID -19. In addition, the data that the Ministry collects is available on the Nippon Hoso Kyokai (NHK) website in the form of Excel files. NHK is a government-owned public broadcasting company. In this study, COVID-19 data on infection cases, mortality, and polymerase chain reaction (PCR) test statistics were collected and incorporated into charts that can be used as a reference in analysing health and economic policies and guidelines implemented during the onset of the pandemic, the 2020 Tokyo Olympics, and the Go to Travel campaign.

Information on health and economic policies was obtained from government agencies such as the Cabinet Secretariat of Japan and NHK. The Cabinet Secretariat of Japan is the government agency that manages the other ministries of the Japanese government, including the Ministry of Health, Labor, and Welfare. To examine the major policy changes, we obtained information from NHK and created a timeline (see Supplementary Information S1) that provides a monthly summary and also uses sources such as the Japan Times. The purpose of the timeline is to compare the COVID-19 epidemiological situation, health and economic policies, and public opinion on these policies during the Abe and Suga administrations. This timeline not only contributed to the comparison with the quantitative table mentioned above, but also clarified the major events in Japanese health policy.

Data from NHK publications are appropriate for this study for several reasons. NHK is the only “designated public institution” among news agencies in Japan’s Framework Act on Disaster Response [27]. Within Japan, it is the only broadcaster that covers all of Japan. It is known in the international community for its impartiality, accuracy, and high-quality reporting [12]. NHK is known as the textbook of global disaster coverage due to its fast and accurate reporting. NHK is also the media source most trusted by Japanese citizens during the pandemic [28]. It is important to note that much of the data used for this study comes from the Japanese government, such as NHK, and therefore the possibility of bias exists. To avoid bias, we objectively refer to numerical data rather than statements made by broadcasters and authors.

After compiling the timeline, we identified the three most important events that impacted public health in the population: the start of the pandemic with the Diamond Princess Cruise incident, the 2020 Tokyo Olympics, and the Go to Travel campaign. For these three events, different types of data such as government reports, newspaper articles, and quantitative data on infection rates were used to trace the evolution of the health situation and the policy-making process related to the events. The implications and results of each event were analyzed to answer our main research question.

## 3. Results

### 3.1. National Overview

As of 11 December 2021, there were cumulatively ~1.73 million COVID-19 cases and 18,368 cumulative deaths in Japan. The case fatality rate was approximately 1.1%. This was lower than the USA at 1.6% and France at 1.5%. However, it was higher than other East Asian countries such as Singapore at 0.3% and South Korea at 0.8% [29]. As for vaccinations, 77.57% of the population, or 97,941,682 people, were fully vaccinated. This equates to 198,014,376 doses administered [29]. The currently approved vaccines in Japan include Takeda/Moderna, Pfizer/BioNTech, and Oxford/AstraZeneca [30].

Japan does not have an expert-led body that can make decisions autonomously in the same way that the Centers for Disease Control and Prevention (CDC) does in the USA or the Korean Centers for Disease Control and Prevention (KCDC) in Korea, but rather bodies that have been given an advisory role [31]. Before 3 July 2020, COVID-19 recommendations were being generated by the Novel Coronavirus Expert Meeting. This advisory board was supervised by the Director of the National Institute of Infectious Diseases, Takaji Wakita. However, as a result of disagreements between public health experts and the government, the government created The New Coronavirus Infectious Disease Control Subcommittee to replace the Novel Coronavirus Expert Meeting. With this new government subcommittee, the Japanese government aimed to improve its coordination and alignment with public health officials [32].

**Figure 1:**
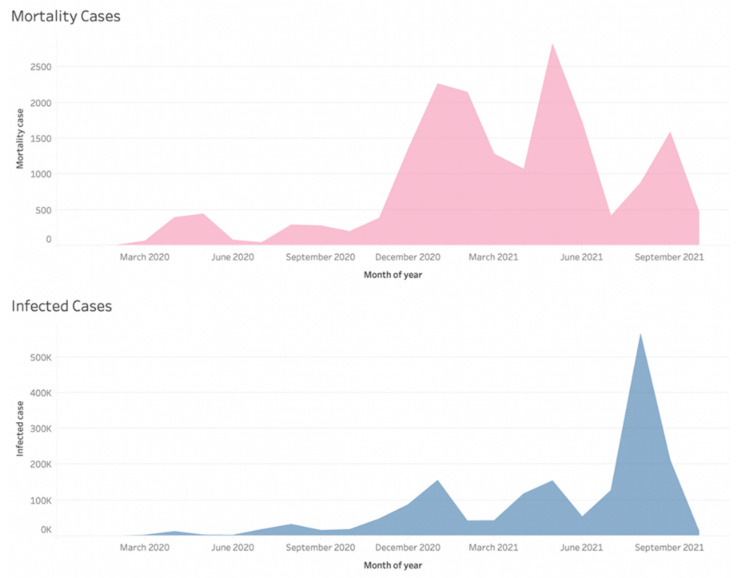
Monthly mortality cases (upper) and infected cases (lower) (Adapted from source NHK data [33]).

**Figure 2:**
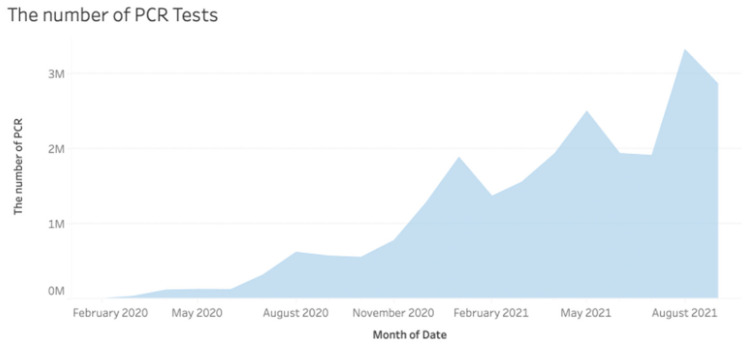
Monthly number of PCR Tests (Adapted from source: NHK data [33]).

An overview of the COVID-19 pandemic in Japan is shown in Figure 1 and Figure 2. Figure 1 shows the infected and mortality cases, and Figure 2 shows the number of PCR tests. In Figure 1, we can see that the mortality rate in April 2020 and May 2020 was higher than in other periods. In contrast, the mortality rate between July 2021 and August 2021 is lower than in other periods. As mentioned in the introduction, the government pushed for increased vaccination before July 2021 in anticipation of the 2020 Tokyo Olympics.

Comparing Figure 1 with Figure 2 shows that the number of infected cases and the number of PCR tests follow similar trends. Furthermore, the number of PCR tests declined before the Olympics and increased after the Olympics ended. The number of PCR tests also declined when Japan announced the “With Corona” policy on 1 October 2020. More detailed information on the policy can be found in Supplementary Information S1.

**Figure 3:**
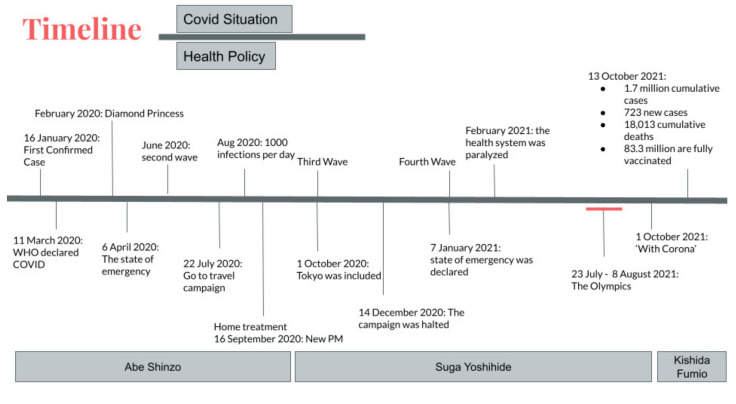
Summarized overview, with timeline up to 13 October 2021.

Figure 3 provides summary information on the dynamics of the pandemic and public policy during the period in question. The description of the pandemic, located on the top side of the figure, shows the most serious public health emergencies, such as waves of the disease. The bottom side shows the public measures taken during the pandemic, such as the “state of emergency”. Finally, the changes of the Prime Minister in the last two years are also shown.

### 3.2. The Initial Response to the Pandemic and the Data Gap

Due to Japan’s geographic proximity to China, there were a significant number of infected cases in early January 2020 [10]. However, official statistical figures show that the number of infected cases was low at the beginning of the pandemic. Explanations for this data gap include a lack of reliability and availability of COVID-19 tests and suspicion of statistical manipulation by the government.

Figure 2 shows that the number of PCR tests is low in January and February. This may have contributed to the seemingly low number of cases at the start of the pandemic. In February 2020, the government was slow to test, as the average number of tests was only 62.4 per day. Furthermore, a few data points were missing from both official and NHK statistics in March and April 2020 [23]. Specifically, statistics for the previously mentioned months were missing from the Excel file provided by NHK. NHK also highlighted the missing data on the page where the Excel file was made available. These missing numbers are a failure in the government’s early monitoring of COVID-19. During this time, the government had not published clear guidelines on actions for citizens, such as testing instructions or quarantine measures. The high mortality rate between February and April 2020 compared to the number of infected cases may support the argument that insufficient testing was conducted early in the pandemic, highlighting the government’s slow response to contain COVID-19.

In addition to the government’s slow initial response, there has also been suspicion that the Japanese government manipulated the number of reported infected cases. Prime evidence for this allegation is the way the situation in the Diamond Princess Cruise ship was handled. Docked at the Yokohama Bay near Tokyo, one of the passengers tested positive for COVID-19 on 2 February 2020. Subsequently, every passenger was tested. According to official statistics from the Japanese government, of the total 3713 passengers and crew on the cruise ship, 712 were confirmed to have contracted COVID-19, and 14 have died. However, the actual number of confirmed cases is estimated to be higher because non-Japanese passengers were allowed to return to their home countries and tested positive upon arrival [34]. A total of 1011 passengers disembarked, but no additional PCR testing was performed upon disembarkation. No additional quarantine was imposed, and passengers were taken to Shinagawa Station, which is one of the main public transportation hubs in Tokyo. This was partly because the Japanese government concluded that there was no evidence of airborne transmission [35]. Passengers continued on public transportation to their final destinations, which were scattered across the country, and may have started community transmission [34].

Of the passengers, 249 showed abnormal symptoms after returning home, and seven of them were confirmed positive [35]. Considering the asymptomatic infections, there may have been more undetected infections. No follow-up investigations into whether they spread the infection in the community have been released. Moreover, the number of cases was not included in the official national statistics because the ship was owned by a foreign country. Therefore, there is no officially recorded accurate information about the number of PCR tests performed or the infected cases.

After the Olympics were postponed, on 1 April, Prime Minister Abe announced that the government would distribute two reusable cloth masks to every Japanese household. Six days later, he declared a state of emergency for Tokyo, Kanagawa, Saitama, Chiba, Osaka, Hyogo, and Fukuoka, to last until 6 May [36]. He highlighted that it was not a lockdown, but that the declaration depended on voluntary compliance and public transportation would still be allowed [36]. In June 2020, the new smartphone application COCOA began to be used for monitoring infected cases, notifying any person who was in close contact with a person infected with COVID-19 [37].

Figure 3 shows that the number of PCR tests slowly increased after the 2020 Tokyo Olympics were postponed. Kenji Utsunomiya, former chairman of the Japan Federation of Bar Associations and organizer of the “Stop Tokyo Olympics” campaign, argued that while the Japanese government claimed it wanted to balance the economy with the imposition of preventive regulations, its actions showed that it had prioritized the economy [38]. Tokyo Medical Practitioners’ The association also stated the possibility of the possible collapse of the medical system with the Olympics [39]. PCR testing was stepped up beginning in July. Tokyo Governor Koike remarked that the increase in the number of positive cases could be explained by the increased number of PCR tests [40].

### 3.3. The 2020 Tokyo Olympics

The 2020 Tokyo Olympics are considered one of Abe’s biggest achievements during his time as prime minister. It was a chance to show Japan’s resurgence after the 2011 Great East Japan Earthquake off the coast of Tohoku [41].

Japan was experiencing the ‘Lost 20 years’ a period which started in 1990, and the Tohoku earthquake had hit the economy badly [42]. Abe was eager to restore the Japanese economy to the level of the second half of the 1980s [43]. In September 2013, Abe gave a speech to the International Olympic Committee (IOC) in which he declared that the Fukushima disaster site was under control and eventually won the bid to host the 2020 Olympics [43]. In a rare occurrence, to express his optimism and passion for the Olympics, Abe showed his informal side by wearing a Super Mario costume during a public conference.

The onset of the COVID-19 pandemic caused trepidation among Abe and his administration. The Olympics were not only an opportunity to showcase Japan’s recovery from the Fukushima nuclear disaster on the world stage, but also a chance to boost the national economy [44]. On 12 March, Thomas Bach, the president of the IOC, announced that the Olympics would go ahead as planned, prompting international criticism [45]. By the end of March 2020, more than 100 countries had imposed some form of lockdown [46]. In mid-March, despite the worsening circumstances of the pandemic, Prime Minister Abe reiterated that the 2020 Olympics would not be affected [47]. Nevertheless, on 23 March, it was decided between Prime Minister Abe and the IOC, along with the Tokyo Metropolitan Government and other concerned organizations, that the Olympic Games would be postponed to 23 July 2021, and the Paralympics to 24 August 2021 [48]. The president of the Olympics pleaded for a two-year postponement, but Abe opposed this because he wanted the Olympics during his term [49]. Abe had attached significant importance to the success of the Olympics because they were an important milestone in his legacy as the longest-serving prime minister [50].

The Tokyo Olympics influenced epidemiological public health in three ways. First, it made the initial response to the pandemic slow and passive in 2020. The Japanese government was reluctant to admit that the epidemiological situation could negatively impact the opening of the Olympics, and seemingly restricted the advocacy for testing, as mentioned in the previous section. Furthermore, the passive approach caused confusion for non-pharmaceutical interventions. For example, fearing that the Olympics would be canceled, Abe attempted to close schools nationwide [17]. This was met with criticism because the notice period was too short, and several local governments and education authorities did not follow this order [51]. Moreover, the postponement of the Olympics was decided in less than 2 weeks after the IOC officially announced that there would be no change to the original schedule [48]. After the postponement was officially decided, the government began to take action to mitigate the pandemic, such as the building of the temporary hospital.

Even after the delay in the Olympics was finalized, the relatively passive response of the national government was inconsistent with that of local governments. The national government’s strategy was based on recommendations rather than hard imposed restrictions. Regarding the economy, small businesses and restaurants were encouraged to remain open and were simply advised to take the necessary precautions. Many of the critics emphasized the Prime Minister’s unwillingness to pressure the business sector to implement hard COVID-19 mitigation rules, particularly with respect to Tokyo [52].

In contrast, Tokyo Governor Koike held an emergency press conference after the number of infections surged to 41 cases in a single day, by far the highest number on 24 March [53]. Governor Koike urged stronger measures to be taken if cases continue to rise. She called on citizens to take voluntary actions to curb the rise of cases [54]. Referring to Figure 3, the number of tests decreased right before and during the Tokyo Olympics (23 July 2021 to 8 August 2021). Considering that every participant in the Olympics had to be tested, the actual number of tests for the domestic population was lower than shown in the graphs.

The Japanese government implemented various policies during the Olympics, such as prohibiting alcohol consumption in metropolitan Tokyo, wearing masks, prohibiting handshaking, and prohibiting parties [54]. During this period, from 12 July to 22 August, Japan experienced the closest version of a lockdown. Repeated breaches of the rules by foreign athletes were noted by the organizing committee and may have contributed to the fifth wave during the Olympics [55]. In August 2021, immediately after the Olympics, Shigeru Omi, the president of the Japan Health Care Organization, expressed that it was evident COVID-19 had arrived at its peak nationwide [56]. The number of cases in August 2021 was 564,179, the highest number of infected persons ever recorded.

Japanese government officials and the Olympic committee argue that the influx of foreigners was not the main cause of the fifth wave [45]. However, public opinion about the Olympics was not positive, given the growing number of cases. Finally, Abe himself did not personally attend the Olympics, and other political authorities also did not attend due to negative public opinion. Members of the LDP party were quoted as saying that they intentionally avoided being photographed at the Olympics to protect the party from negative publicity as this could lead to a loss of voters [44].

Third, the pressure of the Olympics facilitated increased vaccination efforts, which contributed to a lowered fatality rate during and after the Olympics. Amid fears that the Olympics would be canceled, all political parties agreed to place priority on the vaccination rollout. By 16 August, more than half of the population had received the first dose, and 38% of the population was fully vaccinated [57]. In Western countries, vaccination efforts are often politically motivated, but this was not the case in Japan [58]. Vaccination helped reduce mortality rates despite high infection rates and lack of hospital capacity. For more information on the low mortality rate compared to the number of infected cases, see Supplementary Materials. In early August 2020, the government introduced a system based on home-based medical treatment, except for seriously ill patients and people at high risk due to the lack of hospital rooms [59]. In addition, many international visitors came to Japan for the Olympics, but the quarantine areas proved to be a problem.

### 3.4. Go to Travel Campaign

The Go to Travel Campaign, initiated by the national government on 22 July 2020, was a 3.35 trillion-yen program aimed at stimulating the economy by offering domestic travel incentives. The program was characterized by subsidies covering 50% of travel expenses, up to 20,000 yen per person or 10,000 yen per one day trip. Travelers received a 35% discount on accommodation or transportation, and the remaining 15% discount on shopping and dining during the trip [60]. Prior to the initiation of the program, Japan was experiencing its second transmission wave, and one concern when the campaign was introduced was that social distancing would be harder to maintain as a result of traveling [26]. In the first month, over 2 million people participated in the campaign, and by mid-October, the initiative had increased recurring profits by 3.4 trillion yen [61]. A comparative analysis of the travel-associated incidence of COVID-19 cases for the first month of the campaign showed that the incidence related to tourism was almost eight times greater than in the control period of 22 June–21 July 2020. It also highlighted that despite the decline of the second transmission wave, the number of travel-associated cases continued to increase [25].

**Figure 4:**
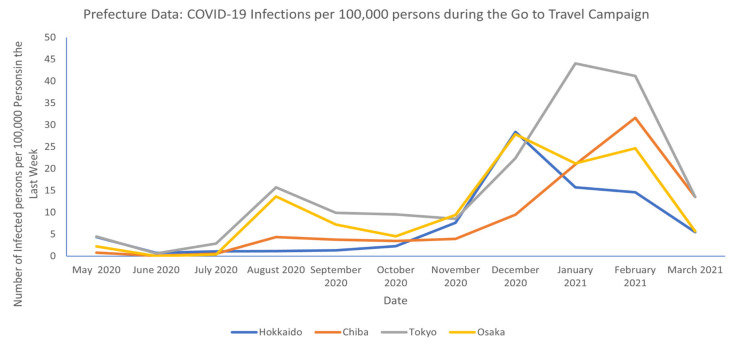
Infections at prefectural level during Go to Travel campaign. (Adapted from source: NHK data [33]).

In 2020, Tokyo, Osaka, Chiba, and Hokkaido were among the top five most visited prefectures for both domestic and international visitors [62]. Figure 4 shows the number of infected persons per 100,000 persons (data points extracted from the 1st of each month) 3 months before and after the Go to Travel Campaign for the previously mentioned prefectures. It can be observed that cases in the prefectures started to increase at the beginning of the campaign, and a decrease in cases is seen two months after the campaign was concluded [33].

By 1 December 2021, the number of daily new case counts have dropped to less than 200 nationwide, and Prime Minister Fumio Kishida plans to resume the Go to Travel campaign due to its sizable impact on the economy [60]. According to a draft of the new plan, the maximum discount per stay will be reduced from 50% of the total cost, the maximum discount amount will be reduced from 14,000 yen to 10,000 yen, and the discount for a day trip will decrease from 10,000 yen to 3000 yen [60]. It is not known to what extent the new plan will help incentivize traveling and boost the economy due to the lower discount rate. Additionally, with the new Omicron variant that is said to be 4.2 times more transmissible than the Delta variant [63], it is difficult to predict whether the new Go to Travel campaign will ever come to life.

## 4. Discussion

Fears of postponing the 2020 Tokyo Olympics resulted in a slow and unstructured initial response to the pandemic by the Japanese government. The onset of the pandemic in Japan began with the government’s lax handling of the COVID-19 situation on the Diamond Princess Cruise ship, which resulted in rapid community infection. However, the effectiveness of quarantine measures in containing the spread of COVID-19 aboard the ship should not be disregarded. Mizumoto and Chowell suggest that following the implementation of the quarantine period, the overall effective reproduction number decreased significantly compared to the value estimated during the early stage of the outbreak [64]. Early spread and fatality mitigation could have been possible by implementing more rigorous detection, testing, and tracing frameworks, but it is important to note that there was little information on the virus at that time. It is also important to note that this situation is unprecedented. While there have been pandemics in recent history, never has this happened in an era of hyperconnectivity in terms of transportation and information.

Early on, political and economic objectives, particularly related to the 2020 Tokyo Olympics, were the most important factors in health policy decisions. As in other countries, there were disagreements between the federal and local governments on the topic of mitigation policies. In particular, municipality leaders felt that the federal policies were insufficient to effectively contain infections. For instance, in the early months of the pandemic, the announcement of school closures in March 2020 was approached in a disparate manner by local prefectures. However, after the decision to postpone the Olympics, the focus on continuing with the Olympics was a motivation for the government to quickly implement policies and programs, such as the country’s COVID-19 app and the vaccine rollout.

The 2020 Tokyo Olympics were held in July and August 2021. Despite the COVID-19 spread mitigation guidelines, the fifth wave began as cases surged during and after the Olympics. However, it is unclear whether the influx of foreigners caused the fifth wave, but it does show that non-pharmaceutical measures during the Olympics were insufficient. Cases did increase, but the Olympics contributed to reducing the mortality rate because of the focus on the vaccine rollout.

Despite the emphasis on the vaccine, Japan experienced a slow rollout. The reason for the slow vaccine rollout is multifaceted. First, confidence in the vaccine in Japan is the lowest in the world, and there is a long tradition of vaccine hesitancy [65]. Japan passed the Preventive Immunization Law in 1948 that mandated vaccination of the population against infectious diseases. However, in 1994, this vaccination requirement was reduced to ‘strongly recommended’ after a few medical incidents thought to be related to combined diphtheria, tetanus, and whole-cell pertussis vaccines [66]. This public fear was recalled in 2013 following media reports of alleged side effects from the human papillomavirus (HPV) vaccine, although the adverse events were later found to be unrelated to HPV vaccination [67].

Second, there was also a shortage of trained medical personnel for vaccination in Japan [21]. Third, Japan has a slower vaccine approval process. The process requires domestic trials to be conducted with Japanese citizens. Finally, the importation of the vaccine was delayed due to a temporary halt in production by Pfizer-BioNTech in the European Union [68]. This highlights that the approval process could be reevaluated in a way that maintains safety but increases efficiency by removing outdated requirements. Prime Minister Suga stated, “The domestic trial requirement isn’t appropriate in an emergency and should be dropped in the future” [69]. It also underscores the need for a reevaluation in the international vaccine supply chain. More efficient vaccine development, approval, and logistics are needed to mitigate economic recession in the case of future pandemics or epidemics.

For example, the Go to Travel campaign proved to be effective in generating revenue from tourism. However, it was observed that during this time, despite the decrease in transmission during the second wave in Japan, there was an increase in travel-associated cases. If the campaign were to resume today, it could achieve similar revenue success, and possibly enjoy a lower number of travel-associated infections, as 77.57% of Japan’s population is vaccinated. There is growing evidence that COVID-19 vaccination reduces the transmission of the virus [70].

Regarding mobility, a study by Kraemer et al. on the effect of human mobility and control measures on the COVID-19 epidemic in China concluded that human mobility predicts the spread and size of epidemics in preliminary stages but is not the sole predictor [71]. The study suggests that after the early stage, variability in daily case numbers was better explained by factors such as local public health response, such as social isolation and hygiene. This finding is consistent with the results of the study by Yamaoka and Oe [72] on the effect of the Go to Travel campaign on spreading COVID-19. Yamaoka and Oe compared the number of deaths during the 41 days of the campaign period with the number of deaths 41 days after the campaign period and found that there was no statistical change in the number of deaths due to COVID-19. They suggest that the campaign “could not be said to have spread the spread of COVID-19” [72]. With increased vaccination rates, non-pharmaceutical interventions, and consistent and correct hygiene practices such as hand washing and social distance measures, the Go to Travel campaign can generate considerable revenue without exacerbating the spread of COVID-19. However, because Go to Travel is a heavily subsidized program, some argue that it is difficult to measure the extent to which it has helped to boost the economy, especially when “those who really want to travel would do so even without the incentive while those who are worried about the risk of infection are unlikely to travel even with the discount” [60].

Overall, prioritization of economic concerns has superseded the implementation of effective COVID-19 infection mitigation strategies. This is also evident in the existent misalignment between government officials and public health experts, who have different priorities in addressing the COVID-19 pandemic. The former believe that the economic consequences should not be disregarded, while the latter urge that effective precautionary measures be enforced to prevent cases from rising. Furthermore, this misalignment is perceived not only at the national level but also among local governments. Japan’s policies put forth during the ongoing COVID-19 pandemic can serve as a case study for Japan and other countries in managing future epidemics and balancing economic health and public health.

### Limitations and Perspectives

As a descriptive analysis, this study is limited by the lack of causal statistical analysis to firmly support the relationship between the policies and guidelines established by the Japanese government and the COVID-19 epidemiological environment. We suggest a statistical analysis of the R rate with the public health policies for further research. Other limitations include difficulties in accessing accurate records of COVID-19 data, especially at the onset of the pandemic. This could be resolved with more direct interaction with the Japanese Ministry of Health, where the data are centralized. We believe qualitative research such as interviews with the relevant personnel involved in each event could also fill some data gaps. As the COVID-19 pandemic is still ongoing at the time of writing, the analysis does not allow us to reach a definitive retrospective conclusion on the overall positive or negative effect of the policies and guidelines implemented by the Japanese government.

## 5. Conclusions

The goal of our study was to examine how the Japanese government has attempted to maintain a balance between the health of the population and the health of the economy. To do this, we used a combination of quantitative data, health and economic policy information from various sources, and data and information on three specific events (initial response to the pandemic, 2020 Tokyo Olympics, and Go to Travel campaign). We conclude that Japan has prioritized its economy over effective COVID-19 health policy, although it has maintained a relatively good balance between the two. Japan’s policies during the ongoing COVID-19 pandemic are an important case study of how delicate dealing with future epidemics and the balance between economic health and public health can be.

So far, despite Japan’s successful management of the disease outbreak, the Japanese government has faced multiple challenges throughout the pandemic. In general, the government prioritized economic vitalization over public health, but understanding that reducing COVID-19 infections and the economy are closely intertwined, the government decided to maintain the day-to-day economic activity, but at a lower intensity. There were no strict lockdowns as in many European countries.

Japan attempted to keep the infection number down by declaring the ‘State of Emergency’, but the government rules were mostly non-binding, being classified as ‘recommendations’. Curbing transmission was dependent primarily on compliance by individuals or municipalities. To accelerate this compliance, the Japanese government introduced a compensation system and repetitively delivered messages about avoiding “3Cs” settings (Closed place, Crowded space, Close contact), while distributing free masks to every national resident.

The initial national response had room for improvement, with domestic political turmoil and a lack of testing. This passive response is heavily linked to the Olympics, which was an opportunity to boost the national economy. The distribution of the masks did not occur until several weeks after the initial incident. Moreover, Japan did not appropriately trace the ‘Diamond Princess Cruise’ passengers, who may have brought the virus to Japan for the first time.

The 2020 Tokyo Olympics had an ambivalent effect on public health. Despite the implementation of the strictest guidelines during the Olympics, the number of cases surged following the Games. However, the government focused on the rollout of vaccines before the Olympics, which contributed to the low fatality rate. The vaccines rolled out did not produce the expected economic results because of the postponement of the Olympics and its no-spectator policy.

The ‘Go to Travel Campaign’ was introduced to increase domestic tourism and boost the national economy. While it boosted domestic travel revenue, it appears to have led to an increase in transmissions and an increased number of infections in the municipalities with popular tourist destinations.

The COVID-19 pandemic is still ongoing, but our descriptive analysis has shown that Japan has been relatively successful in balancing its economic and public health so far. Several lessons can be learnt from the events in Japan that could be helpful in the case of future COVID-19 variants or other viruses. First, the population must have reached a certain level of immunity before stimulus efforts, especially travel, can be launched. Second, contingency plans need to be in place in case the stimulus effort exacerbates the epidemic. Finally, better tracking methods should be developed in the hope of curbing epidemics in their early stages and minimizing economic impact.

## Data Availability

Not applicable.

## Supplementary Materials

The following supporting information can be downloaded at: https://www.mdpi.com/article/10.3390/epidemiologia3020016/s1. (references [18,70,71,72,73,74,75,76,77,78,79,80,81,82,83,84,85,86,87,88,89,90,91,92,93,94,95,96,97,98,99,100,101,102,103,104,105,106,107,108,109,110,111,112,113,114,115,116,117,118,119,120,121,122,123,124,125,126,127,128,129,130,131,132,133,134,135,136,137,138,139,140,141,142,143,144,145,146,147,148,149,150,151,152,153,154,155,156,157,158]).
