# Supplementary material for: Balancing Public & Economic Health in Japan during the COVID-19 Pandemic: A Descriptive Analysis
Source: Epidemiologia (Basel). 2022 Apr 8;3(2):199–217. doi: 10.3390/epidemiologia3020016 (PMC9620936; doi:10.3390/epidemiologia3020016)
Supplement: Supplementary file 1 [file epidemiologia-03-00016-s001.zip › epidemiologia-1577519-Supplementary.pdf]

Timeline from News Articles

1. **Shinzo Abe Period**

The culmination of former Prime Minister of Japan, Shinzo Abe's administration, can be summarized as a three-fold period whereby his crisis supervision, preparation for the Tokyo Olympics and control over the economy were critically put into question [17].

January 2020

On January 15th 2020, Japan confirmed its first case of COVID-19, being the second case of coronavirus in the Asian continent outside of China [73]. The infected patient was a man of Chinese nationality based in the Japanese Prefecture of Kanagawa, who had traveled to Wuhan and acquired a fever on January 3rd after his return to Japan [74]. However, in these early stages of COVID-19 pandemic the Japanese government encountered itself in midst of a growing wave of criticism over its national response, as it did not require citizens coming from Wuhan to quarantine, considering these kinds of obligations to be potentially infringing human rights. It only requested travelers to be mindful of their actions by reporting their health situation and keeping themselves isolated at home or government-provided accommodations for two weeks. Nevertheless, after the reproach, by the end of January, the Japanese government decided that, starting on February 1st, it would refuse the entry of those foreign nationals who would have traveled to the Chinese province of Hubei in the two weeks prior to their arrival to Japan [75].

At the political level, January had also been a turbulent month for Abe's administration featuring recent controversial issues revolving around bribery and the illicit usage of political funds for cherry blossom-viewing parties [75]. With respect to the Japanese economy, the coronavirus outburst implied the start of a series of aggregated challenges for Abe administration's plans, especially in regard to the boosting of inbound tourism [18].

February 2020

February was marked by the Diamond Princess cruise ship incident. On February 2nd, 2020 an 80 year-old passenger from Hong Kong on a Diamond Princess cruise an 80 tested positive first. The ship carried 2,600 passengers and 1,000 crew members. It's roundtrip journey started from the Yokohama Port on January 20th then traveled to Kagoshima, Hong Kong and Okinawa, and returned to Yokohama Port on February 3rd [76]. After this first confirmation, all the passengers and personnel were forced to stay on board, whereby by mid-February, it was announced by the Health Ministry that those older than the age of 80 would be allowed to leave the ship if their tests resulted negative [77]. Nonetheless, the remaining passengers stayed until February 19th, with more positive cases appearing after their disembarkment [76]. On February 27th, it was recognized by Dr. Norio Ohmagari, the director of the Disease Control and Prevention Center at the government's National Center for Global Health and Medicine, that quarantine measures placed on the cruise ship were incomplete [72]. The total number of cases infected on the ship was 712, along with 13 deaths [76].

Furthermore, the government announced to dedicate about ¥15.3 billion of its national expenditure to ease the burdens caused by the demanding situation. As part of the emergency package for alleviating economic threats faced by business owners, ¥500 billion would be devoted to creating new loans for small enterprises, giving special attention to hotels. A total financial aid amounting up to ¥30 million was also offered to national mask producers to secure its provision, expecting them to manufacture 600 million masks over a one-month period [78]. Other policies included the provision of 50 temporary public dwellings by the Tokyo Metropolitan Government [79]. As for overseas operations, the government scheduled five flights to bring Japanese nationals back home, achieving the return of 763 Japanese citizens and their families between the end of January and mid-February [80].

March 2020

By the beginning of March, the number of domestic infections reached 1,000 by March 4th, cumulating those on cruise ships as well [81]. Prime Minister Abe made a public appearance on television on February 27th imploring for joint effort from the citizens to respect the containment measures. He announced that by March 2nd, all schools in Japan would remain closed until the end of Spring vacation, which usually lasted until early April [82]. Contrarily, at the same time, the Health, Labor and Welfare Ministry appealed to nursery schools and after-school day care facilities to persist being open as a way of assisting double-income households impacted by the school closures [83].

The fact that the shutting down of schools was announced in such a relatively short period of time, it was used by many to criticize the Prime Minister's leadership skills as being feeble [84]. Local governments showed a divided response towards the request of school closures, as in Kyoto it was stated that schools would remain open as usual, and in the Ehime prefecture that they would begin closing two days later than the one demanded February 28 [17].

Moreover, from the government's side, it was announced that subsidies were going to be issued to those employees who had to take a temporary leave to be attentive to their children. Also, it would be launched soon a second emergency package utilizing ¥270 billion from its 2019 fiscal reserve fund, in addition to the amount of hospital beds aimed at infected patients being raised from 2,000 to 5,000, and that public health insurance would start covering costs related to COVID-19 testing, as a way of expanding its testing volume [82].

In reference to the Great East Japan Earthquake in 2011, at the sports level, the professional baseball team along with the J.League revealed their decision on postponing the opening of the season [84]. Simultaneously, the government decided that it would coin the current situation experienced as a "historical emergency", a term only attributed to those policy issues that would become historically relevant due to their widespread and severe harm to people's lives, bodies or property [85].

On March 11th, it was officially declared by the WHO that a novel coronavirus pandemic had arisen [86]. Two days later, the Japanese parliament enacted the Act on Special Measures against New Coronaviruses, a legislation that would allow the government to declare a state of emergency regarding COVID-19 [87]. Despite the circumstances, Prime Minister Abe still disclosed by mid-March that the 2020 Olympics would not be affected [87]. Nevertheless, by March 23rd, it was decided between Prime Minister Abe and the International Olympic Committee, along with the Tokyo Metropolitan Government and other concerned organizations that the Olympic Games would be postponed to July 23rd2021 and the Paralympics by August 24th 2021 [48].

At the local level, in the last week of March, Tokyo Governor Koike had to hold an emergency press conference after the surge of infections to 41 cases on a single day, being the highest by far. She urged that stronger measures might be imposed if cases continued rising, therefore she pleaded for citizen collaboration in taking voluntary actions in containing the increase of cases [53].

April 2020

By the start of April, the number of COVID-19 cases amounted to 3,906 [73]. On April 1st, it was publicized by Prime Minister Abe the government plan aimed at distribution of two reusable cloth masks to every Japanese household. Six days later, he declared a state of emergency for Tokyo, Kanagawa, Saitama, Chiba, Osaka, Hyogo and Fukuoka, which would last until May 6th [36]. He highlighted that it was not a lockdown, but rather it would depend on voluntary compliance and public transit would still be allowed [36].

Meanwhile, Tokyo Governor Koike also expressed her disapproval towards this measure taken, as she believed expecting self-restraint from citizens was not sufficient. In addition, small enterprises and restaurants were still encouraged to remain open, only being warned to take the necessary precautions.

Many of the criticisms emphasized the Prime Minister's unwillingness in pressuring the business sector, particularly in regards to its economically significant capital city [52].

On April 15th, the massive growth of case numbers led to the expansion of the emergency declaration to be applied also to Hokkaido, Ibaraki, Ishikawa, Gifu, Aichi and Kyoto, making listing these prefectures as "special prefectures on alert", and eventually covering the whole country [88]. Regarding its cloth mask distribution measure, by April 27th, the Ministry of Health, Labour and Welfare disclosed that more than 400 local governments had denounced they had received cloth masks in unsanitary conditions. It was known that around ¥46.6 billion had been invested in the total costs of mask provision [89].

In economic terms, in the monthly economic report published by the Japanese government for April it was expressed that the economy was experiencing an accelerated decay, being the first time since May 2009, after the Lehman shock, that the term "worsening" had been used in the report [90].

May 2020

On May 6th, 96 people were newly infected, it was the first time since March 30th that the number of newly confirmed infected cases per day nationwide had decreased below 100 [91]. On May 3rd, the Japanese government announced extending the declaration of state of emergency until May 31st, even though Prime Minister Abe assured that if experts gave favourable analysis in regards to the number of COVID-19 cases by May 14th, he would retract the declaration earlier than May 31st. In that event, on May 13th the government lifted the state of emergency in 39 prefectures. Only Hokkaido, Tokyo, Kanagawa, Chiba, Saitama, Osaka, Hyogo and Kyoto were left under the emergency declaration, although Prime Minister Abe also expected their situation to be lifted before May 31st [92].

At the same time, the panel of experts released a new proposal with guidelines that specified the lifting of declaration on a prefecture would be possible under the condition that there was "a total of less than 0.5 new infections per 100,000 people in the past week" [93]. Further to this, three types of prefectures were categorized depending on their spread of infection cases: (i) "Specified Prefectures on Alert", which will have to undergo accordingly to a declaration of state of emergency, (ii) "Prefectures on Alert for the Spread of Infection", meaning measures preventing rise of infections will be reinforced there one step at a time, and (iii) "Prefectures on Alert for the Spread of Infection", which will have to be observed closely whilst distancing measures must be in place [93].

On May 20th, the prefectures of Osaka, Kyoto and Hyogo were lifted from the declaration of emergency [94]. In the same week, on May 24th, Tokyo, Kanagawa, Chiba, Saitama and Hokkaido also had their emergency declaration removed, leading to the state of emergency being fully rescinded [95].

June 2020

The month of June represented the beginning of the Second Wave of COVID-19 in Japan. On June 2nd, the first "Tokyo Alert" was proclaimed by the Tokyo Metropolitan Government given the deteriorating signs of infections, after offices were requested to close on June 1st. Nonetheless, on June 11th, the "Tokyo Alert" was revoked [96].

As for the policies in place, by mid-June, the request for closure was also no longer demanded to the restaurants and entertainment facilities, as long as they guarantee the compliance to prevention guidelines. Events were also allowed to be organized at reduced capacities and the professional baseball games resumed without spectators [97]. Furthermore, a new smartphone application called COCOA began to be used for monitoring infected cases, which would notify any person who would have been in close contact with a person infected with COVID-19 [37].

July 2020

Infected cases kept increasing in July, especially in Tokyo. At the start of the month, the situation seemed to improve with a drop of cases below 100 for the first time in seven days, but on July 9th the number of infections escalated to three digits in one single day, amounting to 224 newly cases, surpassing the 206 cases on April 17th, which had been the highest number recorded until then. Tokyo Governor Koike remarked that the increase in the number of positive cases could be explained by the enlargement of PCR tests, even though precaution still had to be prioritized [40]. Days after, July 14th represented one of the tipping points of the infection situation in Tokyo, rising to the "Infection spread alert", the most severe of the four levels of alert [98]. On July 15th, it was recorded the highest number of infected people in one day nationwide since April 10, exceeding 600 infections 5 [99]. The Prefecture of Kanagawa also issued a "Kanagawa Alert" on July 16th, after observing their newly infected cases raised to more than 40 [100].

On July 21st, the chairman of the Japan Medical Association, Mr. Nakagawa stated that if the rapid accumulation of infected cases persists, with the further drastic increase there could be a potential collapse of the medical care delivery system, including also normal medical care systems. He insisted that Japanese residents should avoid traveling in the four consecutive holidays [101]. Notwithstanding, by July 22nd, Japan's domestic travel subsidy campaign named Go To Travel was installed to revitalize the country's tourism industry and stimulate demand for its economic activities, under the condition that necessary precaution measures were respected [102].

August 2020

By mid-August, at the Infectious Diseases Society of Japan, chairman of the subcommittee on countermeasures against new coronaviruses, Mr. Shigeru Omi, expressed that it was evident COVID-19 had arrived at its peak nationwide [56]. By the end of the month of August, newly infected people were being urged to be hospitalized according to the Infectious Diseases Control Law. Nonetheless, it heightened the burden on public health centers, as more asymptomatic and mildly ill patients were being admitted [59]. Thus, days after, those who did not show symptoms or had lighter symptoms started being treated at home [59].

In addition, a new policy was released by the government which planned to secure the medical supply system, broaden the inspection system to 200,000 cases a day, and guarantee the provision of vaccines to all citizens by the first half of the following year [103]. Nevertheless, Prime Minister Abe's serving period came to an end by mid-September.

2. Yoshihide Suga Period

From September 16th, 2020 to October 4th, 2021, Yoshihide Suga served as Prime minister of Japan and the Liberal Democratic Party president. This section looks at COVID-19 measures and COVID-19 policies for the Tokyo 2020 Olympics during Suga's administration.

September 2020

In September, the total of newly infected COVID-19 cases was 15,194. The new daily cases remained stable throughout the month with a daily average of around 506 new cases a day. The government focused on establishing a basic countermeasure system and trying to balance infection control and socio-economic activities this month. On the 15th, the government decided on spending more than 1.6 trillion yen from the reserve fund to expand the testing system, 739.4 billion yen to secure beds and accommodation facilities, and 4.3 billion yen to assist medical institutions in maintaining PCR test kits [104]. The government also relaxed restrictions on the size of crowds at professional sports matches, movie theatres, and other events to 50% of the full capacity [105]. The Go To Travel campaign continues normally. However, those who did not feel well were suggested to not go out [106].

October 2020

There were a total of 17,744 new COVID-19 cases in October. In general, the trend of new cases shows a slight increase with an average of 572 new cases. Despite the increasing trend, the government put an emphasis on economic revitalization and implemented policies such as allocating additional budget for Go To Travel so that all operators can maintain a 35% discount [107]. The Tokyo Metropolitan Government had an independent incentive for Tokyo residents traveling in Tokyo, as participants could receive a maximum of 25,000 yen per person per night with a total budget of 2.2 billion yen Go To Eat also had an allocated budget of 61.6 billion yen to incentivize people to dine out [107]. To further recover the economy, the government relaxed immigration restrictions so that from October 1st, foreigners with medium- to long-term status of residence are allowed to enter Japan [108]. In terms of COVID-19 policies, The Ministry of Health, Labor, and Welfare also signed a contract with Moderna to supply 25 million people with vaccines if Moderna succeeded in developing a COVID vaccine [109]. Additionally, the Ministry of Health, Labor, and Welfare decided to not charge for vaccination so those who want to be vaccinated can receive it free of charge [110]. Regarding the Olympic Games, the government and the Games Organizing Committee confirmed the policies to prevent infection during Olympic Games. Measures such as restricting the athlete activity range and singing in the locker room and thorough disinfection and ventilation of rooms would be implemented [111]. Temperature measuring stickers are a new technology that changes color in response to body temperature. This new technology was intended to be used as a temperature measurement alternative during inspection to save time [112].

November 2020

A total of 47,501 new COVID cases were added in November, more than two and half times the number of new cases last month. A steady increasing trend is observed from the case data in November, with a daily average of 1,583 new cases. A cluster of infections was observed in Hokkaido, and as a result, the alert stage was raised from 2 to 3. The government asked restaurants to shorten the business hour to 10 pm, and a maximum of 5 people is allowed when dining out [113]. The government also requested people to restrain from going out unless it is urgent, and this intensive measure was decided in effect from November 7th to 27th [114].The government also decided to continue Go To Campaigns such as Go To Travel and Go To Eat despite the case surge at the beginning of the month. However, on November 22nd, the government gave in to the rapid increase of cases, and Prime Minister Suga suspended Go to Eat and Go to Travel reservations to destinations where the infection had spread on, and people living in Sapporo and Osaka were asked to refrain from traveling until December 15th [115]. Regarding the Olympic Games, the government and the Organizing Committee were trying to decide the maximum number of people to be accommodated and considered a quarantine exemption for foreigners [116].

December 2020

There were a total of 86,925 new COVID-19 cases in December. There were 2804 new daily cases on average, and a steady increasing trend in daily cases was observed. Due to the severity of the spread of the infection and a rapid increase in critically ill patients, the Osaka prefecture announced the ‘Medical Emergency Declaration’ at the beginning of the month to ask people to refrain from going out for two weeks [117]. The government also stopped new foreigners from entering Japan from December 28th due to the presence of the new mutant strain [118]. To address the lack of funds required for Go To Travel, the government decided to spend more than 380 billion yen from reserve funds to Go To Travel [119]. Osaka was still excluded from Go To Travel, and Go To Travel was eventually decided to stop operations nationwide from December 28th to January 11th, 2021 [120]. In terms of Olympic Games measures regarding COVID-19, the government planned to spend 100 billion yen on COVID-19 measures for the Olympics [121]. The Organizing Committee also established the Infectious Disease Control Center for athletes. The postponement of the Olympic Games was estimated to cost about 200 billion yen to the Organizing Committee and the Tokyo Metropolitan Government [121].

January 2021

There were a total of 154,988 new COVID-19 cases in January. There were almost 5,000 new daily cases on average, with a steep increase in the beginning and a steady trend of small decrease as the time passed. To address the sudden increase of infection, Prime Minister Suga issued a state of emergency from January 7th to February 7th, targeting Tokyo, Saitama, Chiba, and Kanagawa prefectures [122]. On January 9th, it was stated that immigrants must submit a mandatory inspection certificate before departure to Japan [123]. However, on January 13th, the government suspended the entry of foreigners altogether [124]. On January 20th, Japan signed a contract with Pfizer Inc. with enough vaccines for 72 million people [125]. Go To Travel and Go To Eat remained suspended.

February 2021

The government tried to work on infection prevention in February. A total of 41,896 new COVID-19 cases were added in February, and the daily new case average is about 1496 on average. In comparison with January, the overall total and daily new cases decreased significantly, and the overall trend showed a steady decrease in new cases. A total of 10 prefectures are under the state of emergency, including Tokyo, Osaka, Aichi, Fukuoka, Kanagawa, Saitama, Chiba, Hyogo, Kyoto, and Gifu the state of emergency will be in effect until March 7th [126]. Penalties for businesses that do not comply with COVID-19 government measures were established in Special Measures Law as a countermeasure against COVID-19 [127]. Pfizer COVID vaccine was officially approved, and pre-inoculation began for approximately 40,000 frontline health workers [128]. By the end of the month, six out of the ten prefectures, including Osaka, Hyogo, Kyoto, Kansai, Aichi, Gifu, and Fukuoka, were lifted out of the state of emergency [129]. Go To Travel and Go To Eat remained suspended. For the Olympics, the prevention control guideline was published, in which it asks athletes to take PCR tests once every four days until the Olympic Games are finished [130].

March 2021

A total of 42,450 new COVID-19 cases were added in March, and the daily new case average is about 1369 on average. Despite having a lower daily new case average, both the total new case and daily new case trend increased. The state of emergency was extended for Tokyo, Saitama, Chiba, and Kanagawa until March 21st, and major restaurant chains shortened their business hours to 8 pm [131]. The Cabinet decided to spend more than 2 trillion yen as a reserve fund for the new COVID-19 measures, such as preventing food and drink infections [132]. Go To Travel and Go To Eat remained suspended [133].

April 2021

A total of 117,700 new COVID-19 cases were added in April. The daily new case average is about 3923 and had a steady increasing trend this month. Compared to March, April showed a significant increase in the total new case number and daily new case average, possibly as a result of the cancellation of the state of emergency in multiple prefectures. Saitama, Chiba, Kanagawa, and Aichi continued to be under priority measures to prevent infection spread, and the government also encouraged people to refrain from traveling in the upcoming Golden Week holiday in May [134]. Suga declares the third state of emergency in Tokyo and three western prefectures of Osaka, Kyoto, and Hyogo, effective from April 25 to May 11 [135].

May 2021

There were a total of 153,724 new COVID-19 cases in May, with a daily average of about 4959 new cases. Although there was a downward trend in new cases, the overall new case number and average daily new case number both increased significantly compared to April. The government extended the state of emergency in Tokyo, Osaka, Hyogo, and Kyoto from May 11th to May 31st, and Aichi and Fukuoka were added to be under the state of emergency from May 12th [136]. Hokkaido, Okayama, and Hiroshima were further added to be under a state of emergency on May 16th [137]. Okinawa was also under a state of emergency from May 23rd to June 20th [138]. Following Okinawa, on May 28th, all nine

prefectures mentioned above re-extended the state of emergency to June 20th [139]. The government also accelerated vaccination to control infection. In terms of Olympic Games measures regarding COVID-19, the Olympic Games Committee requested overseas coaches and ministers to refrain from visiting their athletes [140].

June 2021

There were a total of 53,097 new COVID-19 cases in June, with a daily average of about 1770 new cases. The trend seemed to show a steady decrease in new daily cases. The Cabinet decided to issue a vaccine passport in July [141]. The state of emergency was lifted in the ten prefectures except for Okinawa, and priority measures are still held in seven prefectures, including Tokyo and Osaka [142]. Vaccination started for 1,600 athletes and instructors participating in the Tokyo Olympics free of charge [146]. A policy is made to allow up to forty people in the leader class from each country to visit the Olympic Games [144]. Chiba first canceled public viewing for the Olympic Games, then the Tokyo Metropolitan Government also canceled public viewing and some venues were converted for vaccination purposes [145, 146].

July 2021

A total of 126,607 new COVID-19 cases were added in July, with a daily new case average of 4084. As the state of emergency was lifted in multiple prefectures and the Tokyo Olympic games started, the number of new infections increased sharply in July, and Japan entered its fifth wave of nationwide infection [55]. The government issued a fourth state of emergency to Tokyo from July 12th to August 22nd, and Okinawa would extend its emergency state till August 22nd as well [147]. As the Olympic game started on July 23rd. Saitama, Chiba, Kanagawa, and Osaka were once again brought to be under a state of emergency, along with Hokkaido, Ishikawa, Hyogo, Kyoto, and Fukuoka [147, 148]. The Tokyo Metropolitan Government established a support base for health center operations as a countermeasure for any occurrence of COVID-19 cases in the Olympic Village [149]. The tournament committee in Hokkaido decided to hold games without any audience or spectators. Athletes infected with COVID-19 were allowed to return to the games after completing a ten-day quarantine or two negative PCR tests 24 hours apart within six days [150].

August 2021

New COVID-19 cases kept on increasing in August. There were a total of 564,179 new cases, with a daily average of 18,199 new cases. At the beginning of the month, the government established a system based on home medical treatment except for critically ill patients and people at high risk [151]. Priority Measures to Prevent Spread applied to Fukushima, Ibaraki, Tochigi, Gunma, Shizuoka, Aichi, Shiga, and Kumamoto were added from the 8th to the 31st as a countermeasure against COVID-19 [152]. However, as the infection escalated, Ibaraki, Tochigi, Gunma, Shizuoka, Kyoto, Hyogo, and Fukuoka were brought under a state of emergency from August 17th to September 12th [163]. On August 25th, Hokkaido, Miyagi, Gifu, Aichi, Mie, Shiga, Okayama, and Hiroshima, were further to the target areas of the state of emergency, and Saga, Nagasaki, and Miyazaki were added to be under priority measures [154]. However, despite the surging case number, the government relaxed the maximum number of immigrants to enter Japan from 2,000 to 3,500 a day [155]. The government explained that because the Tokyo Olympics ended there would be room available for quarantine [155].

September 2021

In September, a total of 210,613 new cases were added, with a daily new case average of 7,020. September new infection cases showed a significant downward trend. The state of emergency in 19 prefectures was extended to September 30th from the 12th, and the prefectures of Miyagi and Okayama were shifted to priority measures [156]. As a result, 19 prefectures were under a state of emergency,

and 8 prefectures were under priority measures [157]. Additionally, the number of people who received the second vaccine shot was estimated to exceed 50% [158].
